# Strategies to sustain and scale up youth friendly health services in the Republic of Moldova

**DOI:** 10.1186/1471-2458-13-284

**Published:** 2013-03-28

**Authors:** Venkatraman Chandra-Mouli, Valentina Baltag, Luwam Ogbaselassie

**Affiliations:** 1 Department of Reproductive Health and Research, World Health Organization, 20 Avenue Appia, Geneva 27, CH-1211, Switzerland; 2 Department of Maternal Newborn Child and Adolescent Health, Adolescent Health and Development, World Health Organization, Geneva 27, Switzerland; 3 Dalla Lana School of Public Health, University of Toronto, Toronto, Canada

**Keywords:** Youth friendly health services, Youth friendly health centres, Quality of care, Scaling up

## Abstract

As part of a multifaceted effort to respond to the needs of young people more effectively, the Ministry of Health of the Republic of Moldova established pilot Youth Friendly Health Centres (YFHC) in 2001.

In 2005, after 12 YFHC were set up and implemented, the MOH identified that while they were serving a useful function, four problems remained needed to be addressed – the lack of an operational definition of the term youth friendly health services, the lack of objective data on the added value of the existing YFHC, the low coverage of the existing YFHC and the almost complete reliance on donor agencies for funding the effort.

The MOH addressed each of these problems systematically. While challenges still exist, the MOH has taken important steps to ensure that all young people in the country can obtain the health services they need.

## Background

When the Republic of Moldova was part of the Union of Soviet Socialist Republics and during the ten years following the collapse of the Union, the focus of health service provision in country was on delivering a defined set of - mainly - curative health services to the population as a whole [1].

Young people were required to seek care from doctors providing health services to all segments of the population in their catchment areas. However, some specialised services were available - health units in educational institutions and adolescent health offices in some polyclinics. The main focus of these units was to screen young people for health problems and provide curative care [1].

The tumultuous societal changes that occurred in the 1990s led to a deterioration of the health status of all segments of the population. Young people were especially affected. Deaths from injuries, trauma (including self-inflicted trauma) and intoxications; and levels of STI including HIV, early pregnancy and mental difficulties and disorders all rose alarmingly [2].

The health system was not geared to respond to the rapidly changing needs and problems of young people. Even as importantly, young people were not willing to seek care because they did not trust or like it. The Ministry of Health of the Republic of Moldova (MOH) realized the pressing need to reform the health system to make it respond more effectively and sensitively to young people [2].

With the support of the United Nations Children’s Fund, the MOH opened three pilot youth-friendly health centres (YFHC). The first two clinics were set up in Chisinau, the capital city in 2001 and 2002. The third one was set up in 2003 in Stefan Voda. All three clinics were housed in existing community-based health facilities. Some medical and support staff from the health facilities they were housed in, were seconded to them on a full-time or part- time basis. The clinics provided health services in addition to – and complementary to – the standard set of health services being provided in the community-based health facilities. They included preventive sexual and reproductive health services, and responses to adolescent-specific physical problems such as acne and psychological ones such as body-image concerns. They guaranteed privacy and confidentiality, and provided nonjudgemental care in a comforting environment. In 2005, the MOH set up nine more YFHC across the country, with support from the International Development Agency, the World Bank, and the Swiss Development Cooperation Agency [2].

No formal assessment of the work of the YFHCs was carried out. But from the reports that both the funding bodies and the MOH received, they had a positive impression of their work. Further, through discussions with development partners and participation in regional meetings, they were aware that similar efforts were under way in other countries of the region, and were broadly supportive of them.

The MOH recognized that there were four challenges in building on the initial gains of the 12 YFHC and developing a nationwide initiative. First, apart from a small circle of people directly associated with the 12 YFHC, most health workers in the country were not clear about what youth friendly health service provision meant. Second, even though anecdotal reports suggested that the 12 YFHCs were successful in attracting young people and responding to their needs, there was no objective assessment of this. Third, the 12 YFHC had at best a modest coverage. This meant that a large proportion of young people in the country had no access to these services. Finally, the MOH was concerned about the sustainability of the 12 YFHCs, given that donor funding was for a limited period.

The problem of promising pilot projects dying a natural death when the funding comes to an end is a real one, in many countries and in many fields of public health including adolescent health [3,4].

## The Ministry of health’s efforts in moving from twelve pilot projects to nation-wide implementation

### Defining, standardizing and placing youth friendly health service provision in Moldova’s public health context

#### Activities undertaken

In order to create a wider shared understanding of the attributes of youth friendly health services, the MOH developed a document titled the National Concept on Youth-Friendly Health Services in the Republic of Moldova [5]. The document defines Youth Friendly Health Services (YFHS) as *“…services that correspond to the needs of young people related to accurate and updated information and that offer a full range of accessible services and qualitative care to young people in the most appropriate way. These include an environment that guarantees privacy and confidentiality, as well as services offered by experts free of prejudices and trained in the field of adolescent health and development and youth friendly approaches, for young people to be able to make free and informed choices regarding their health and sexuality“.* It also lists a set of basic principles underpinning the provision of YFHS.

Realizing the need to go beyond articulating the guiding principles and a definition of the concept, the MOH developed standards specifying the required quality of health service provision at each health facility [6]. The input, process and output criteria to accomplish each standard were outlined, as were the actions required to implement and monitor them.

The quality standards document defined adolescents as individuals aged between 10–19 years, and young people as those aged between 10 and 24 years. It stated that while all young people would be targeted, special efforts would be made to reach young people who are particularly vulnerable e.g. those living on the streets and those – especially from the rural areas - left behind by parents who have gone abroad. It outlined packages of basic and specialized services to prevent and respond to developmental problems, nutritional problems, sexual and reproductive health problems, mental health problems and problems resulting from violence. Finally, it listed the health facilities where the basic and specialized health services would be provided.

#### Results achieved

The Concept paper on YFHS helped define and explain what this term meant, and specified their place in the overall national effort to improve the health and welfare of young people in the country. The aim of providing health services to young people in a friendly manner was clearly stated: *“to increase the access of young people to information and qualitative health services as well as other services that correspond to the definition and criteria of ‘youth-friendly’“*. The National quality standards subsequently developed by the MOH provided clear definitions of the quality of service provision that would be required in each health facility, the means by which this was to be done and the means by which quality standards and activities to achieve them were to be measured.

By defining and standardizing youth friendly health service provision, the MOH contributed to ensuring that every health worker in the country could understand what this concept meant and what each would need to do to make his/her health facility youth friendly.

### Demonstrating the results achieved by the 12 YFHC, and documenting the evolution of these centres

#### Activities under taken

The definition of measurable quality standards provided the basis for demonstrating the utility of the YFHC. Building on this, the MOH took two concrete steps. Firstly, it decided to assess the quality of health service provision of the 12 YFHC against the approved standards. It supported the adaptation of the World Health Organization’s generic Quality Assessment Guidebook [7]. The tools developed included guides to interview health facility managers, health service providers, YFHC users and young people in the community, and a check list to observe the YFHC. The quality of health service provision was assessed in 20 health facilities - 12 YFHCs, two women’s health centres and six reproductive health offices. In this exercise, 277 young people (98 health facility users and 179 young people from the communities served by the assessed health facilities), 74 service providers, and 20 facility managers were interviewed [2]. Secondly, the MOH supported the preparation of a case-study to document the process of development of YFHC in the Republic of Moldova and to share the lessons learned. The case study was published in 2010 in a WHO Regional Office for Europe publication *Youth friendly services and policies in the European Region*[2]*.*

#### Results achieved

**Figure 1:**
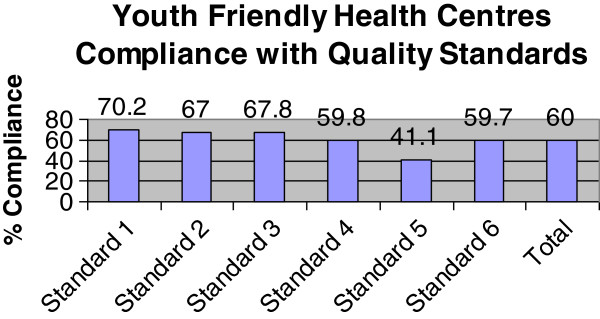
**Compliance of the 12 Youth Friendly Health Centres in Republic of Moldova with the national quality standards (listed below). Standard 1:** Young people know when and where to ask for health services. **Standard 2:** Young people have easy access to the health services they need, when they need them. **Standard 3:** Health service providers maintain the confidentiality and respect the privacy of young people. **Standard 4:** Health service providers mobilize the community (to promote youth friendly health services). **Standard 5:** Health service providers offer effective and comprehensive services in line with young people’s needs. **Standard 6:** All young people have equal access to health services.

The assessment of the quality of the twelve YFHC, the Women’s Health Centres and the Reproductive Health Offices provided the MOH with objective information on the breadth and depth of the compliance with national quality standards of the assessed facilities. They also pointed to where further quality improvement was needed. Figure 1 compares the compliance of each of the 12 YFHC with the national quality standards. The highest levels of compliance were with standards 1–3. The relatively low scores for standard 5 (the quality of case management) pointed to where pressing corrective actions were needed. By assessing the quality of the YFHC against national quality standards, and demonstrating their utility, the MOH provided the grounds to support their continuation.

The detailed analytic case study (referred to above) described the process by which the YFHCs were set up and evolved over time. It also described the barriers that were identified and overcome, and the opportunities that were put to use along the journey. Finally, it also pointed to what needs to be done to build on what has been achieved. Thus it provides a valuable ‘how to’ for the managers of the many health facilities in the country who start work in this area.

Interest in youth friendly health service provision had hitherto been limited to a small number of government-run health facilities and NGOs. The case study provided a tool to inform engage all health professionals in the country to include young people in their thinking and work.

### Preparing a strategy to scale up youth friendly health service provision across the country

To expand the coverage of health services, the MOH drew upon WHO’s guidance [8] to develop a scaling up policy and strategy [9]. The scale-up strategy aimed to engage and build the capacity of available health service providers to expand the provision of health services to young people across the country. The proposed delivery system involving primary and secondary level providers, linked to a centre of excellence is as follows:

i. A network of primary level providers who would inform, educate, and provide primary-level counselling and health services and refer young people who are more vulnerable than others, or require specialized services, to the secondary level providers.

ii. A network of secondary level providers who would deliver similar services at the primary level to adolescents, especially to vulnerable ones who have been referred to them by the primary level. If patients need more specialized care, they would refer them to government-run social welfare agencies and non-governmental organizations that provide additional services.

(Note: Primary level providers would include family doctors (around 2000), school nurses (around 1000, and staff in 32 outpatient units of district hospitals). Secondary level providers include staff from the 12 existing YFHC, the 47 existing Reproductive Health and the three existing Women’s Health Centres).

iii. Staff at both primary and secondary levels are to receive managerial and technical support from the MOH.

#### Results achieved

The scaling up strategy developed by the MOH sets out a stepwise approach to take youth friendly health service provision from 12 centres in urban areas to literally hundreds of existing primary and secondary level health facilities across the country. To do this, the MOH aims to draw upon the expertise of these centres and others in the capital city and around the country to support family doctors, school nurses and staff in the outpatient units of district hospitals to change the way in which they and their health facilities work with and serve young people. The reform of school health services and referrals that was initiated by the MOH in 2008 with WHO’s support is especially relevant here. With a high primary and secondary school enrolment ratio, school health services personnel in the country are well placed to ensure high coverage with a basic package of youth friendly health services.

### Securing funding for expanding the provision of youth friendly health services across the country

To ensure the sustainability of the centres, the MOH advocated with the National Health Insurance Company (NHIC) to take on their funding. The clear definition of packages of health services to be provided at different levels as outlined in the Concept document (referred to earlier) responded to the information needs of the NHIC and made the inclusion of YFHC in the NHIC list easier [10].

This landmark decision was made in 2008. Given that the YFHC had relied entirely on donors for their operations, this was a major step forward but one that needs to be safe guarded and built upon because of the NHIC’s policy to review its decisions on which health services to cover on an annual basis.

#### Results achieved

The quality standards and the accompanying measures of their achievement have been incorporated into several key national health policy documents [10]. This has served to firmly anchor the YFHS provision as a key component of the wider national public health effort. As a result of this, YFHS provision will be sustained in the Republic of Moldova whether or not there is donor funding for this in the future.

## Future challenges

### Scaling up to be managed

The rationale for scaling up health services is clear – to reach young people who were not currently being reached by the 12 existing YFHCs, and to engage a range of primary level providers to deal with the general population of young people, and to redirect the efforts of the YFHCs to reach out to those young people most at risk of health and social problems. The proposed delivery system includes a network of family doctors, school nurses and outpatient units in district hospitals, and a more specialized secondary level which consists notably of 47 Reproductive Health Offices. All these individuals and institutions are to receive managerial and technical support from the MOH as discussed.

The challenges of doing this are also clear – a range of health service providers in the capital city and across the country who are not currently working with/serving adolescents will need to take this up, their capacity will need to be built and they will need ongoing support. In addition, further institutional arrangements to build their capacity and to provide them with ongoing support will need to be set up.

### Funding arrangements for youth friendly health service provision to be safe guarded and expanded

In 2008, the NHIC took on the payment for health services provided to young people by the 12 existing YFHC. Further, bilateral and multilateral donors have agreed to support the scale up effort. These are both positive steps; however there is still more work to be done to secure adequate financial support for youth friendly health service provision in the country on a long term basis.

Firstly, in terms of adequacy, the funds provided by the NHIC does not fully cover the costs incurred the YFHC. Each has to find some top-up funding from elsewhere. Monitoring data on the nature of health services provided and the populations of young people reached, will be needed in the annual reviews of the NHIC which become progressively more rigorous. Secondly, the financing is based on a *per consultation* principle. The more patients a health worker sees, the more the financial return. This is not good for all patients, and certainly not good for adolescents presenting with sensitive conditions; because without adequate time, good rapport building, good history taking and examination, and education and counselling are difficult to do. Thirdly, the NHIC does not make a provision for information and education work either within the health facility or through outreach; an activity that was critical to the success of YFHC.

## Conclusions

In many countries, non-governmental organizations are in the forefront of efforts to provide youth friendly health services. Most of these efforts are small in scale and of limited in duration. The MOH has a key stewardship role to play in enabling youth friendly health service provision to become an integral part of large scale, sustaining programming.

Following the introduction and implementation of 12 YFHC in the Republic of Moldova, the MOH identified problems that could hinder their continued existence and their replication. It worked with technical agencies to address these problems - it defined and standardized the principles and practices of youth friendly health services and placed them in the country’s public health context; assessed the quality of youth friendly health service provision and documented their implementation and evolution; developed a strategy to scale up the provision of health services to young people across the country and to integrate youth friendly health service provision in the national health system; and finally, it took the first steps to secure the long-term financing of youth friendly health service provision.

While challenges exist in managing the scaling up and in securing long term financial support, the MOH has moved ahead well in ensuring that all young people in the Republic of Moldova can obtain good quality health services that they need.

## Abbreviations

MOH: Ministry of Health Republic of Moldova; NHIC: National Health Insurance Company; YFHC: Youth Friendly Health Centres.

## Competing interests

The authors declare that they have no competing interests.

## Authors' contributions

CM conceived the paper and prepared the first draft. VB helped to fill the gaps and to strengthen the paper. LO worked with CM to finalize the first draft of the paper that was submitted to BMC Public Health. CM and VB prepared the revised second draft, in response to the reviewers’ comments. All authors read and approved the manuscript that was submitted for consideration.

## Authors' information

VB and CM are both WHO staff members. CM is based in WHO's headquarters. Till November 2012, VB worked with WHO's European regional office, when she moved to WHO’s headquarters. Both CM and VB have contributed actively to the evolving work in Moldova. VB, who is a Moldovan national, has as intimate understanding of country because she studied and worked there. She also led the documentation of her country's evolving story on the progress of Youth Friendly Health Centres, and has come into contact with all key stakeholders through this process. LO is a student who interned in WHO in 2012.

## Pre-publication history

The pre-publication history for this paper can be accessed here:

http://www.biomedcentral.com/1471-2458/13/284/prepub
